# Preclinical Studies Comparing Efficacy and Toxicity of DNA Repair Inhibitors, Olaparib, and AsiDNA, in the Treatment of Carboplatin-Resistant Tumors

**DOI:** 10.3389/fonc.2019.01097

**Published:** 2019-11-12

**Authors:** Nirmitha I. Herath, Nathalie Berthault, Sylvain Thierry, Wael Jdey, Marie-Christine Lienafa, Françoise Bono, Patricia Noguiez-Hellin, Jian-Sheng Sun, Marie Dutreix

**Affiliations:** ^1^Institut Curie, PSL Research University, CNRS, INSERM, UMR 3347, Orsay, France; ^2^Université Paris-Sud, Université Paris-Saclay, CNRS, INSERM, UMR 3347, Orsay, France; ^3^DNA Therapeutics, Evry, France; ^4^Onxeo, Paris, France

**Keywords:** DNA repair inhibitors, carboplatin, AsiDNA, efficacy and toxicity, resistance

## Abstract

**Purpose:** Carboplatin is used to treat many cancers, but occurrence of drug resistance and its high toxicity remain a clinical hurdle limiting its efficacy. We compared the efficacy and toxicity of DNA repair inhibitors olaparib or AsiDNA administered alone or in combination with carboplatin. Olaparib acts by inhibiting PARP-dependent repair pathways whereas AsiDNA inhibits double-strand break repair by preventing recruitment of enzymes involved in homologous recombination and non-homologous end joining.

**Experimental Design:** Mice with MDA-MB-231 tumors were treated with carboplatin or/and olaparib or AsiDNA for three treatment cycles. Survival and tumor growth were monitored. Toxicities of treatments were assayed in C57BL/6 immunocompetent mice. Circulating blood hematocrits, bone marrow cells, and organs were analyzed 10 and 21 days after end of treatment using flow cytometry and microscopy analysis. Resistance occurrence was monitored after cycles of treatments with combination of AsiDNA and carboplatin in independent BC227 cell cultures.

**Results:** Olaparib or AsiDNA monotherapies decreased tumor growth and increased mean survival of grafted animals. The combination with carboplatin further increased survival. Carboplatin toxicity resulted in a decrease of most blood cells, platelets, thymus, and spleen lymphocytes. Olaparib or AsiDNA monotherapies had no toxicity, and their combination with carboplatin did not increase toxicity in the bone marrow or thrombocytopenia. All animals receiving carboplatin combined with olaparib developed high liver toxicity with acute hepatitis at 21 days. *In vitro*, carboplatin resistance occurs after three cycles of treatment in all six tested cultures, whereas only one became resistant (1/5) after five cycles when carboplatin was associated to low doses of AsiDNA. All selected carboplatin-resistant clones retain sensitivity to AsiDNA.

**Conclusion:** DNA repair inhibitor treatments are efficient in the platinum resistant model, MDA-MB-231. The combination with carboplatin improves survival. The association of carboplatin with olaparib is associated with high liver toxicity, which is not observed with AsiDNA. AsiDNA could delay resistance to carboplatin without increasing its toxicity.

## Introduction

For decades, the hallmark of cancer treatment has been cytotoxic treatments such as radiotherapy and chemotherapy. Combination therapy, a treatment modality that combines two or more therapeutic agents or medical devices, is the new cornerstone of cancer therapy. The association of several anti-cancer drugs enhances efficacy compared to mono-therapy protocols as it targets key complementary pathways reducing the occurrence of drug resistance, while simultaneously providing therapeutic anti-cancer benefits. The “Achilles heel” of many new promising combination treatments is the emergence of multidrug resistance and high toxicities that were not anticipated from single treatment protocols. Carboplatin is a platinum-containing chemotherapy drug used to treat many cancers including ovarian and lung cancers. Though largely prescribed, its high toxicity may induce cessation of treatment or arbitrary dose reductions, which may potentially compromise patient outcome (1). Carboplatin acts by forming toxic drug–DNA adducts, and its toxicity has been correlated to the level of these adducts present in healthy tissues that limit the dose tolerated (2). For many malignancies, patients initially respond to platinum but then develop acquired resistance. The cellular response, which confers resistance to carboplatin, is multifactorial and poorly understood (3, 4). It has been observed that the intracellular mechanisms by which cells become resistant to carboplatin include increased drug detoxification by the thiol groups in metallothionein and glutathione, improved tolerance to nuclear damage, and DNA repair, leading to a concomitant reduced accumulation of intracellular carboplatin and reduction in apoptosis. DNA repair systems and anti-apoptotic factors have been identified as potential mechanisms underlying platinum-based treatment resistance. Thus, impairing mechanisms of DNA repair with repair inhibitors could provide a way to overcome resistance (4).

A dramatic shift toward therapies targeting DNA repair has occurred in the last decade with the discovery of PARP inhibitors. The most advanced in this field, olaparib (AZD2281 or KU-0059436), a specific inhibitor of Poly (ADP-Ribose)-polymerase 1 and 2 (PARP), has been successfully used in the context of synthetic lethality in the treatment of tumors with *BRCA* mutations, as monotherapy as well as in combination with other chemotherapy agents (5). Significantly, increased risk of hematologic toxicities was observed for patients treated with PARPis combined with single-agent chemotherapy (5). The efficacy of PARPi on platinum-resistant tumors (6–8) gave hope that combination of PARPi with platinum-based treatments would both improve tumor control and prevent emergence of resistance. However, clinical experience with therapies combining PARPi with chemotherapies has been, in general, mixed. For example, combining olaparib with carboplatin and paclitaxel chemotherapies in the clinic has been challenging due to myelosuppression, and reductions in the full single-agent doses of all drugs had to be undertaken to decrease the toxicity (9, 10). Therefore, there is a need to develop novel therapeutic strategies targeting DNA repair with lower toxicity and to test how combinations of DNA repair inhibitors and carboplatin can help to fight carboplatin resistance.

**Figure 1:**
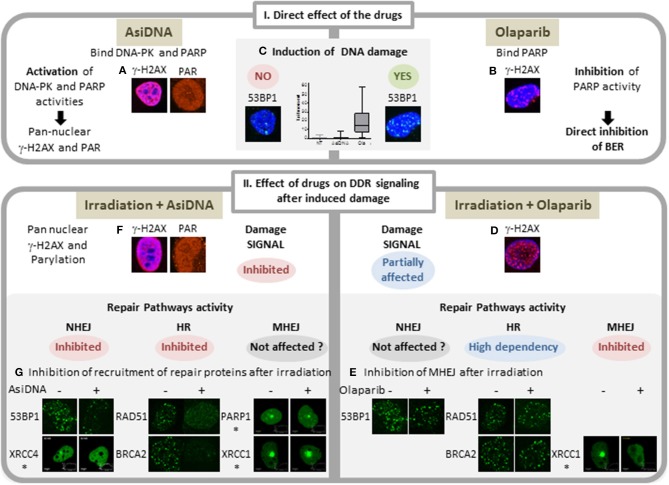
Comparison of main features of AsiDNA and Olaparib activity on DNA repair. I: Activity of the inhibitors AsiDNA (left) and olaparib (right). AsiDNA is a short modified DNA mimicking double-strand break. It binds DNA-PK and PARP enzymes and activates their kinase and polymerase activity leading to modification of a large number of cellular proteins including pan nuclear γ-H2AX protein and poly-ADP-Ribose (PAR) **(A)**. These modifications occur in absence of DNA damage as revealed by 53BP1 foci and comet assay **(C)** (12). In contrast, olaparib inhibits PARP polymerase activity and induces increase of DNA damage (13) **(B,C)** probably through inhibition of base excision repair (BER) and increase of replicative stress. II: Drug effect on damage signaling and recruitment of DSB repair proteins after damage. Damages were induced either by γ irradiation or laser (^*^). Three DSB repair pathways were monitored: homologous recombination (HR), non-homologous end joining (NHEJ), and micro homology end joining (MHEJ, also called alt-NHEJ). Whereas, olaparib inhibits the formation of foci of XRCC1 and PARP1 (14), it has no effect on formation of radio-induced foci of γ-H2AX, 53BP1, RAD51, and BRCA2 **(D,E)**. In contrast, AsiDNA inhibit recruitment of 53BP1, XRCC4, RAD51, and BRCA2 (15) **(F)** and do not prevent recruitment of PARP and XRCCI **(G)**.

We have developed a novel DNA repair inhibitor AsiDNA, which has already undergone two Phase I clinical trials [DRIIM (11); DRIIV-1, NCT03579628 in progress], with no evident toxicity in patients. These molecules act differently to usual inhibitors used in medicine such as PARPi. Instead of blocking catalytic activity of their targets, AsiDNA promote their activation (Figure 1). AsiDNA are short modified DNA molecules that bind DNA-dependent protein kinase (DNA-PK) (15, 16) and PARP (17) and activate, respectively, their kinase and polymerase activity leading to modification of numerous proteins in the cell. DNA-PK and PARP activation by AsiDNA triggers a false signal of DNA damage in the absence of DNA injury and prevents further recruitment of DNA repair enzymes on damaged chromosomes (Figure 1). Consequently, the DNA repair enzymes are diverted from their primary objective, the double-strand breaks on chromosomes, which results in inhibition of their repair and ultimately cell death. Clinical and preclinical studies have demonstrated that this strategy sensitizes tumors to DNA damaging treatments such as radiotherapy (11, 18). In this work, we compare the ability of AsiDNA or olaparib to potentiate carboplatin treatment in a breast cancer model resistant to platinum.

Due to the increasing concerns with toxicity of combined treatments, modern clinical trial designs will need to incorporate translational studies, which may be used to guide patient selection, drug scheduling, and treatment response. We used immunocompetent animal models to investigate the efficacy and the toxicity of the combination of AsiDNA or olaparib with carboplatin.

## Materials and Methods

### Ethics Statement

All animal experimentation was approved by the local authorities and was carried out in strict accordance with the European Union guidelines for animal care. Only animals with unobjectionable health were selected to enter testing procedures. Animals were routinely monitored including mortality checks, assessment of animal welfare and tumor growth by observation, control of feed, and water supply.

### Experimental Animals

MDA-MB-231 cell-derived-xenografts (CDXs) were obtained by injecting 5 × 10^6^ cells into the mammary fat pad of 6 to 8-week-old adult female nude NMRI-nu Rj:NMRI-Foxn1^nu^/Foxn1^nu^ mice (*Janvier*). The animals were housed at least 1 week before tumor engraftment, under controlled conditions of light and dark (12–12 h), relative humidity (55%), and temperature (21°C). When engrafted tumors reached 80–250 mm^3^, mice were individually randomized into groups of 8–10 to different treatment groups. Two sets of experiments including four groups of treatment were performed to analyze combinations of carboplatin with AsiDNA or carboplatin with olaparib. Tumor growth was evaluated three times a week using a caliper and calculated using the following formula: (length × width × width)/2. Mice were followed for up to 3 months, and ethically sacrificed when the tumor volume reached 1,500 mm^3^.

Toxicity studies were performed in 6–7-week-old C57BL/6 mice (Janvier, France). Non-tumor-bearing mice were used to allow long-term studies and monitor potential reversion of adverse effects. Animal weights were noted prior to treatment and were routinely monitored for signs of weight loss (daily during treatment and three times a week for the remainder), signs of discomfort, diarrhea (including examination of sawdust), abnormal/rapid breathing, generalized clinical signs of blood loss including pallor of skin, necrosis of the tail and toe nails, and any other signs of abnormal behavior. Groups of three animals per treatment were sacrificed at days 7, 10, and 21 after end of the last treatment to analyze bone marrow and organ toxicity.

### Drug Administration

Treatments were administered for three cycles comprising 1 week of treatment followed by 2 weeks of rest (Figure 1A). AsiDNA (Agilent, USA) was administered for 4 consecutive days through intraperitoneal (IP) injection, at daily doses of 5 mg (Figure 1). Olaparib (AZD-2281, Roowin chemicals, France) was administered at daily doses of 200 mg/kg through oral gavage (PO) for 5 consecutive days. Carboplatin was administered as a single IP injection of 80 mg/kg for toxicity studies or 50 mg/kg for efficacy studies alone or 4 h after AsiDNA or olaparib treatment on day 1 for each cycle. The first day of the first treatment cycle was designated day 0.

### Toxicity Study

#### Complete Blood Count (CBC) Analysis

Blood samples (>80 μl) were harvested through submandibular bleeding without anesthesia in EDTA tubes, since isoflurane is known to interfere with CBC data. The samples were collected prior to treatment, day 7 post-2nd cycle, and days 7 and 21 post-3rd cycle of treatment. CBC analysis was performed using a 3-part-diff automated hematology cell counter-MS9-3s according to manufacturer guidelines (Melet Schloesing Laboratories). In this analysis, the total white blood cell count (leukocytes), and three main cell types within this population (lymphocytes, monocytes, and granulocytes), the total red blood cell count (erythrocytes), the mean corpuscular volume (MCV), hematocrit, hemoglobin, and thrombocyte (platelet) levels were acquired.

#### Nucleated vs. Non-nucleated Cells

To assess the ratio between nucleated and non-nucleated cells in the peripheral blood, non-nucleated cells were lysed using an acetic acid (3%) solution with methylene blue (Stem Cell Technologies, France), and the nucleated cells were manually counted.

#### Bone Marrow Harvesting

Murine bone marrow cells were harvested 7 and 21 days post-3rd cycle of treatment. The femurs were isolated from mice post-sacrifice and cleaned of any remaining muscle. The femurs were ground in 2 ml of ice-cold PBS using a mortar and pestle. Once the bone marrow was visible in PBS, the supernatant was transferred to a 50-ml falcon tube through a 40-μm cell strainer. The strainer was rinsed with an additional 1 ml of PBS. The typical yield of bone marrow cells from both femurs per non-treated C57BL/6 mouse was approximately between 100 and 150 million.

#### Peripheral Blood and Bone Marrow Smears

For peripheral blood smears, 4 μl of fresh blood was placed on the frosted edge of a glass slide and smeared across using another glass slide. For bone marrow smears, ~10 × 10^6^ bone marrow cells obtained from the femur were centrifuged at 1,500 rpm for 10 min at room temperature. The cell pellet was resuspended in 30 μl of RPMI-50% FBS. Fifteen microliters of this cell suspension was placed on a slide and smeared across using a second glass slide.

#### Sternal Imprints

The sternum was harvested and cut lengthwise using a scalpel blade. Imprints were performed directly by pressing the cut side of the sternum firmly on to a glass slide.

#### Wright–Giemsa Staining of Bone and Peripheral Blood Smears and Sternal Imprints

Wright–Giemsa (Electron Microscopy Sciences, USA) labeling was performed on peripheral blood smears, bone marrow smears, and sternal imprints according to the manufacturer's instructions. The Wright–Giemsa-stained sections were subjected to a blinded analysis by an experienced anatomical pathologist for all the treatment groups.

#### Histology of Tibia and Other Organs

Both ends of the tibias were clipped in order to permit the entry of the fixative. The tibias were fixed in 4% PFA, decalcified, and paraffin embedded. In addition, other organs such as the lung, liver, kidney, and the brain were also harvested and fixed in 4% PFA and paraffin embedded. All organs were sectioned and stained with H&E for further histological analyses by an experienced pathologist.

#### Harvest of Splenocytes and Thymocytes

The spleen and thymus were harvested post-sacrifice. The tissues were ground through a 40-μm cell strainer (BD falcon) using a plunger from a syringe with 2 ml of ice-cold PBS, until only stromal tissue remains.

#### Flow Cytometric Assessment of Bone Marrow, Spleen, and Thymus

Erythroid, lymphocyte, and myelocyte lineages of the bone marrow were assessed using flow cytometry. The erythroid lineage was assessed using TER119 and CD71, the lymphocyte lineage was assessed using B220 and Thy1.2, and the myelocyte lineage was assessed using Gr-1 and Mac1 antibodies. Splenocytes were stained using B220 and Thy1.2 antibodies while thymocytes were stained using Thy1.2. Antibodies are listed in Table S1.

### Cell Lines and Resistance Selection

The MDA-MB-231 cell line was purchased from the ATCC. The triple-negative breast cancer BC227 cell line was derived from a patient sample in the Laboratory of Preclinic Investigation of the Institut Curie. Cell lines were verified by short tandem repeat profiling (Geneprint 10, Promega) at 10 different loci (TH01, D21S11, D5S818, D13S317, D7S820, D16S539, CSF1PO, AMEL, vWA, and TPOX) and tested negative for *Mycoplasma* contamination with the VenorGeM Avance Kit (Biovalley). For repeated cycles of treatment, cells were treated as described previously (12). Briefly, cells were seeded in six-well-culture plates with 2 × 10^4^ cells per well and incubated 24 h at 37°C before addition of the drugs (2.5 μM AsiDNA, 7 μM carboplatin). Cells were harvested on day 6 after treatment, washed, and counted after staining with 0.4% trypan blue (Sigma Aldrich, St. Louis, USA). Cells were seeded in six-well-culture plates, and allowed to recover for 6 more days. Another cycle of treatment/recovery was then started for up to five cycles. In a control of non-treated growth, similar cycles of dilutions were performed in medium without addition of AsiDNA. Survival was calculated at the end of the treatment week of each cycle by the ratio of living cells in the culture with drug treatment on the number of living cells in the culture without the drug. Resistant clones (called RC1-6) were selected out of independent populations that received five cycle treatments with carboplatin.

### Statistical Analysis

Statistical analyses were performed with StatEL software (ad Science). Mann–Whitney test was performed to analyze variations between groups in the toxicity studies. Survival curves were plotted according to the Kaplan–Meier method, and survival fractions of mock-treated and treated groups were compared using log-rank test analyses. *P*-value of less than or equal to 0.05 was considered to be a significant difference.

## Results

### Efficacy of Combination Treatments

We analyzed the efficacy of olaparib and AsiDNA alone or in combination with carboplatin treatment in *nude* mice xenografted with the MDA-MB-231 triple-negative breast cancer model. These cells are homologous recombination proficient and were found to be moderately sensitive to olaparib and AsiDNA, *in vitro* [Table 1 in (12)]. The dose of 50 mg/kg carboplatin was chosen as no apparent toxicity or weight loss was observed at this dose in animals (Figure S1). Carboplatin was administered every 3 weeks to recapitulate a fractionated treatment commonly used in the clinic. Olaparib and AsiDNA were administered daily during the weeks of carboplatin treatment. Three cycles of treatment with single agent administration or in combination with carboplatin were performed. There was no observed toxicity at these doses in any treatment group.

**Figure 2:**
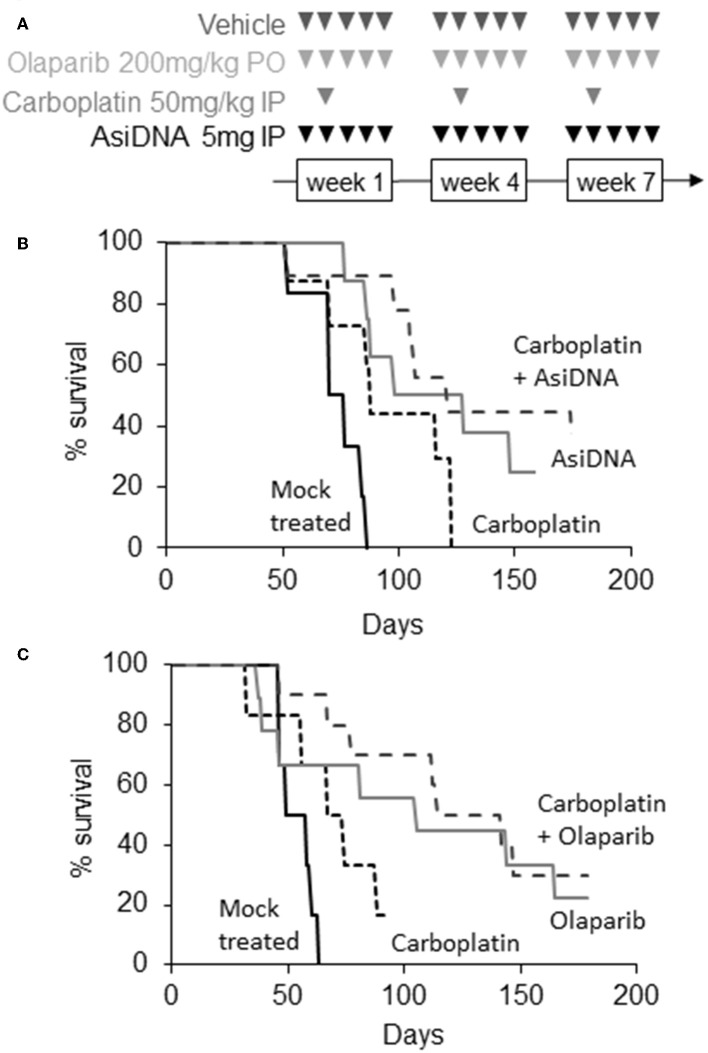
Efficacy of combination treatments. **(A)** Scheme of different treatments, triangles indicate days of treatment; **(B,C)** Kaplan–Meier representation of two independent experiments. Survival was calculated as the fraction of living animals with tumor size <1,500 mm^3^; **(B)** mock treated (*n* = 6, black), treated with carboplatin (*n* = 8, small dotted black), AsiDNA (*n* = 8, gray), or combination of AsiDNA and carboplatin (*n* = 10, large dotted black); **(C)** mock treated (*n* = 6, black), treated with carboplatin (*n* = 6, small dotted black), olaparib (*n* = 9, gray), or combination of olaparib and carboplatin (*n* = 10, large dotted black).

In agreement with published data (19), we found that MDA-MB-231 tumors are highly resistant to platinum treatment (Figure 2). In the two independent experiments, carboplatin treatment had no significant effect (*P* > 4 × 10^−2^) with a median increase in survival of 11–16 days. Olaparib and AsiDNA monotherapies increased median survival by 43 and 51 days, respectively (Figure 2). However, the heterogeneity in animal responses limited the significance of the difference (*P* = 5.2 × 10^−2^ for olaparib and 5.2 × 10^−2^ for AsiDNA; Table S2). To further examine response after repair inhibitor treatments, we analyzed the tumor growth profile in groups separated into responder (survival > 100 days; *n* = 5) and non-responder (survival <100 days; *n* = 5; Figure S1). Interestingly, with both inhibitors, a significant decrease in tumor volume was observed after 28 days, corresponding to the end of the second cycle of treatment. Combination treatments associating DNA repair inhibitors (olaparib or AsiDNA) with carboplatin showed the most significant efficacy (*P* = 0.0003) (Figure 2; Table S2).

### Toxicity of Combined Treatments in Blood Cells

**Figure 3:**
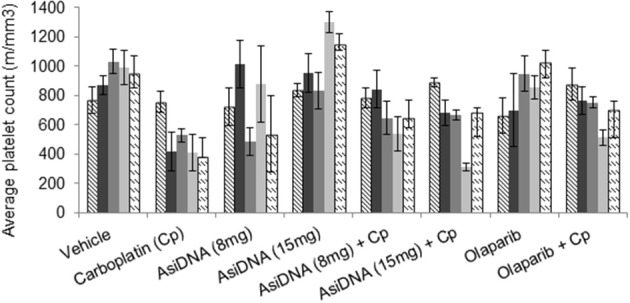
Blood toxicity. Different combinations of carboplatin 80 mg/kg, with AsiDNA 8 or 15 mg or olaparib 200 mg/kg were used to treat mice. Counts of platelets before treatment (hatched), 7 days after beginning of first cycle (dark gray), 7 days after second cycle (dark gray), and 7 or 21 days after third cycle (light gray and dashed, respectively).

The treatment protocols were performed in immunocompetent C57BL/6 mice to improve detection of carboplatin toxicity as the carboplatin-containing regimens induced significant thrombocytopenia (reduction of platelets) and leukopenia in patients. Blood samples were taken pre-treatment, 7 days after the first cycle, and 10 and 21 days after the end of the three-cycle treatment. Monotherapies with olaparib or AsiDNA were well-tolerated (Figure S2) and did not show any toxicity in blood cells or bone marrow. As already reported in patients treated with carboplatin, we observed a significant deficit in blood and bone marrow cells (erythroid and lymphocyte lineage) that was reversed on day 21 before the second cycle (Figure S3). A significant decrease in the platelet count was observed (*P* < 0.01) 7 days post-carboplatin treatment (Figure 3). This decrease was observed after the first injection and did not increase significantly with additional cycles. It showed only partial reversion 21 days after end of treatment. Association of AsiDNA or olaparib with carboplatin did not increase the deficit in blood cells (Figure S3). Interestingly, the combination of carboplatin with the DNA repair inhibitors stimulated the complete reversal of the drop in platelets at later times (Figure 3).

**Figure 4:**
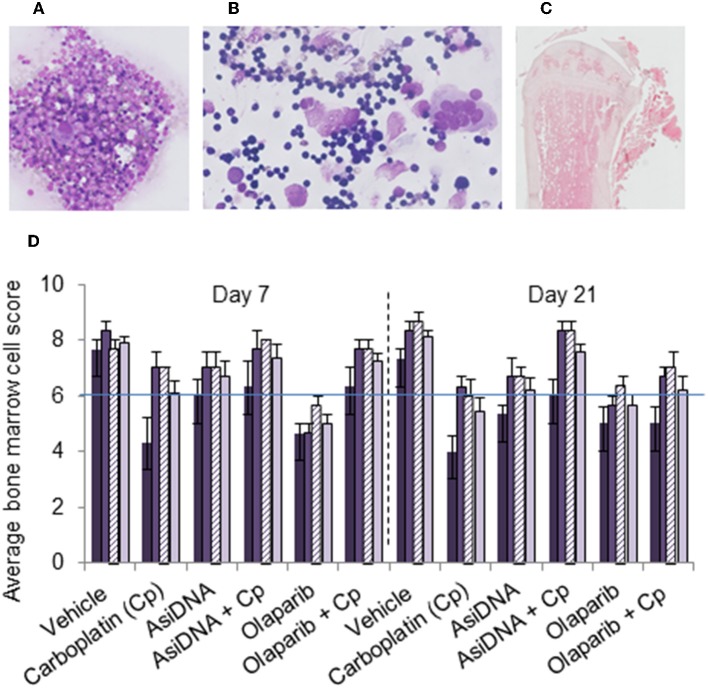
Bone marrow myelogram. Wright–Giemsa staining of **(A)** sternal imprints and **(B)** bone marrow smears and **(C)** H&E staining of paraffin-embedded tibia. **(D)** Mean values of bone marrow myelogram (six animals) treated with various treatments (indicated in abscissa) and sacrificed at day 7 or day 21 after the third cycle. Samples were scored from 0 to 10 according to the following scale: poor (0–3), moderate (4–7), or good (8–10) indicating high, moderate, or no toxicity in erythroid (dark violet), myeloid (medium violet), and megakaryocyte score (dashed violet), and density (light violet).

A single administration of carboplatin induced acute thymic atrophy, with reversal at day 21, and was reproduced with no significant changes at each cycle (Figure S4). Flow cytometry confirmed a significant reduction of the number of T lymphocytes and erythroid or lymphocyte lineage in bone marrow after each cycle of treatment. This effect was not enhanced by AsiDNA and appeared to be partially prevented by olaparib. No significant differences were noted in the bone marrow cells between treatment groups receiving carboplatin with or without AsiDNA or olaparib (Figure S4). In order to further examine the bone marrow cellular morphology, density, and the presence of all cell lineages, histology samples were subjected to a blinded analysis by an experienced anatomical pathologist for all the treatment groups. The samples from animals treated with carboplatin revealed a moderate to high toxicity, which was not significantly enhanced by AsiDNA or olaparib (Figure 4).

### Distribution of AsiDNA in Bone Marrow and Organs

**Figure 5:**
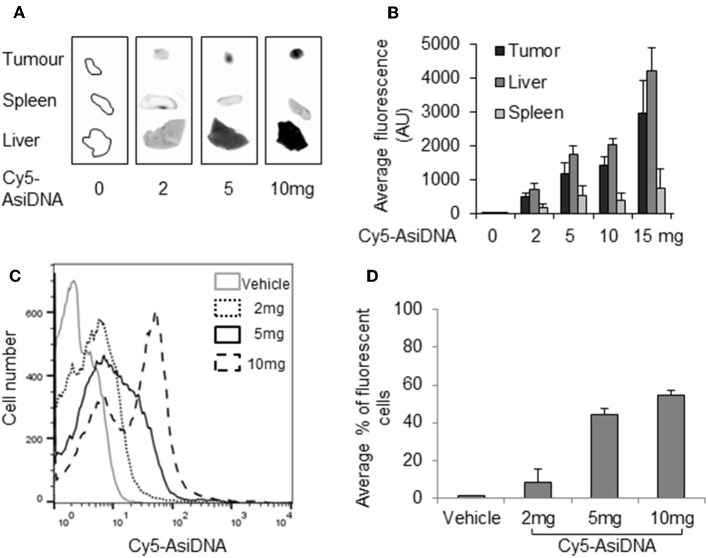
Distribution of AsiDNA. Animals were injected with cy5-AsiDNA (0, 2, 5, 10, 15 mg) and sacrificed 6 h later, and fluorescence was monitored in organs: tumor, liver, and spleen: **(A)** characteristic pictures and **(B)** mean fluorescence quantification for six animals. **(C,D)** Bone marrow was sampled and analyzed by flow cytometry: **(C)** representative distribution of bone marrow cells of a mouse from each treatment group and **(D)** mean percentage of fluorescent bone marrow.

As the lack of increase in bone marrow toxicity could be due to the poor addressing of AsiDNA to this location, we checked the uptake of fluorescent cy5-AsiDNA by flow cytometry of femur bone marrow. The uptake was dose dependent and more than 60% of the cells were fluorescent 4 h post-administration of AsiDNA-cy5 (Figures 5C,D). Moreover, the analysis of the other organs showed that AsiDNA molecules administered by IP injection efficiently reached the spleen, the liver, and grafted tumors located in the fat pad (Figures 5A,B). As observed for most drugs, the highest uptake of AsiDNA was in the liver.

### Liver Toxicity of the Combination of Olaparib With Carboplatin

**Figure 6:**
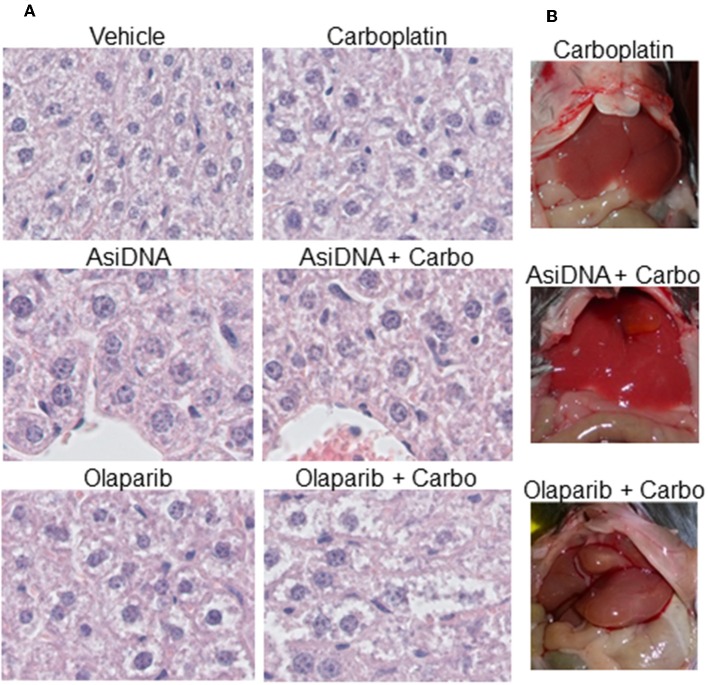
Hepatomegaly in mice treated with a combination of olaparib and carboplatin. **(A)** Example of histology samples of liver of mice exposed to various treatments, **(B)** macroscopic view of treated livers.

No organ toxicity was revealed after macroscopic analysis of mice treated with monotherapies or the combination of carboplatin with AsiDNA. Though liver uptake is high, AsiDNA did not enhance carboplatin toxicity in this organ. However, all mice treated with olaparib in association with carboplatin presented with hepatomegaly (enlargement of the liver; Figure 6). This was already evident in 1/3 mice 7 days post-treatment, and all animals presented with an abnormal liver 21 days post-treatment. Microscopic analysis confirmed that the livers of all the animals treated with a combination of olaparib and carboplatin presented with hepatocyte edema, pre-necrotic hepatocytes, bi-nuclear hepatocytes, and infiltrating lymphocytes (Figure 6A). Bi-nuclear hepatocytes are suggestive of a highly regenerative liver, whereas infiltrating lymphocytes indicate inflammation. Symptoms such as edema, pre-necrotic hepatocytes, and infiltrating lymphocytes were not observed in other treatment groups. The onset of this side effect worsened between 7 and 21 days following end of treatment and it is difficult to predict whether this could be reversed at a later time.

### Inhibition of Acquired Resistance to Carboplatin by AsiDNA

**Figure 7:**
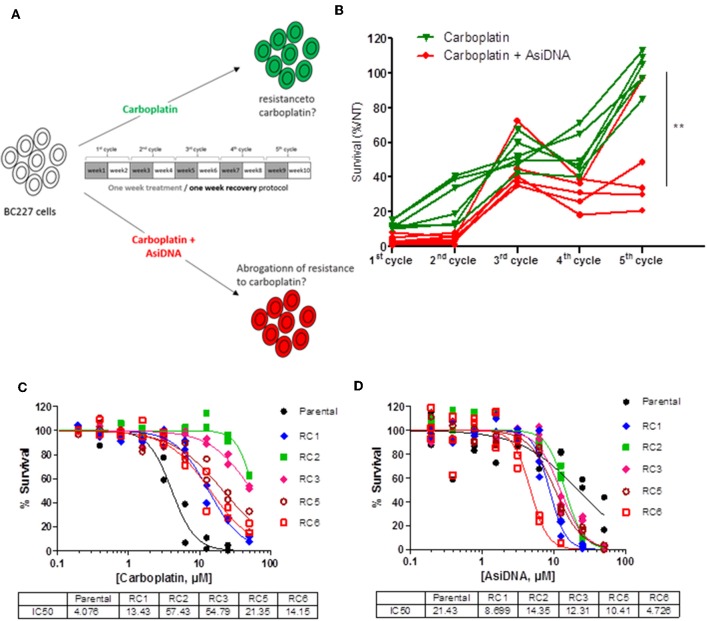
Effect of AsiDNA on the emergence of resistance to carboplatin. Cells were treated for 1 week and grown without treatment for an additional week for one to five cycles. **(A)** Schedule of treatment. Gray square: weeks of treatment with 7 μM carboplatin ± 2.5 μM AsiDNA; white square: weeks without treatment. **(B)** Survival to each cycle of treatment was monitored in 10 independent BC227 populations treated with either carboplatin (green triangles) or carboplatin + AsiDNA (red circles). **(C,D)** Survival of carboplatin-resistant populations (RC) vs. parental cells after 1 week treatment with **(C)** carboplatin or **(D)** AsiDNA. Survival was estimated by the ratio of treated cells on non-treated cells after 1-week treatment. ^**^P < 0.01.

The combination of AsiDNA and carboplatin being well-tolerated, we tested how the combined treatment could prevent emergence of carboplatin resistance during treatment. To monitor resistance occurrence, we used the BC227 triple-negative cell line, which is highly sensitive to carboplatin. We performed cyclic treatment protocols that have been shown to facilitate resistance emergence to PARPi but not to AsiDNA (12). Five to six independent populations were treated for five cycles comprising 1 week with carboplatin combined or not with AsiDNA followed by 1 week of recovery (without drug) per cycle. Survival of the BC227-independent populations treated with carboplatin increased progressively with cycles of treatment up to complete resistance at the 5th cycle for all the independent cultures (Figure 7B). In contrast, most populations receiving AsiDNA concomitantly with carboplatin showed increase to 40% survival at the 3rd cycle that did not change with the following cycles of treatment. Only one population among the six treated with the combined molecules was 100% resistant at the 5th cycle whereas the four others showed similar survival at the 3rd cycle (Figure 7B). These data suggest that AsiDNA could inhibit or delay the emergence of carboplatin resistance in BC227. We purified clones from the carboplatin-resistant populations and analyzed their sensitivity to AsiDNA and to carboplatin. As expected, all the clones were more resistant to carboplatin than the parental cell line. Surprisingly, all of them showed a higher sensitivity to AsiDNA, indicating that the major pathway to promote resistance to carboplatin is involved in sensitivity to AsiDNA (Figures 7C,D).

## Discussion

Preclinical studies with animal models allow the preparation of clinical protocols and, to some extent, the anticipation of adverse effects of new treatments. In this study, we tested the hypothesis of using DNA repair inhibitors olaparib or AsiDNA to treat carboplatin-resistant tumors. The carboplatin protocols reproduced the usual schedule used in patients of one injection every 3 weeks. The DNA repair inhibitors olaparib and AsiDNA were given daily (open days) during carboplatin treatment. Olaparib association with platinum has been tested in several clinical trials and revealed a moderate efficacy associated with an increased toxicity. Though olaparib is usually administered continuously, it has been shown that intermittent dosing of olaparib ameliorated some of the toxicity, allowing its successful administration with paclitaxel and carboplatin in previously untreated recurrent ovarian cancer patients (20). We used this combination as a control in our study to evaluate the efficacy and the toxicity of the association of AsiDNA with carboplatin in a triple-negative breast cancer mouse model, resistant to carboplatin. AsiDNA and olaparib both act by inhibiting DNA repair albeit through very different mechanisms. Olaparib binds to PARP (21) and prevents base excision repair (22) and alternative non-homologous end joining pathways (23). AsiDNA binds PARP and DNA-PK and stimulates their activities asynchronously to the detection of the damage to be repaired (15). The signal generated by AsiDNA prevents recruitment of repair enzymes involved in homologous recombination and non-homologous recombination (Figure 1). Both molecules sensitize tumor cells to radiation (18, 24).

The MDA-MB-231 tumor cells are moderately sensitive to olaparib and AsiDNA (25). These tumor cells are proficient for homologous recombination (26) and therefore can repair the replication block due to the inhibition of single-strand break repair by olaparib (27). AsiDNA, having a broader mechanism of action that leads to the inhibition of non-homologous end joining and homologous recombination, seems to have a higher antitumor effect than olaparib. The activity of both repair inhibitors takes time to be effective as responding tumors appear to maintain the same rate of growth for the first 3 weeks. After the second cycle, half the treated tumors stopped growing or regressed (responder tumors). No correlation was identified between the size or the initial growth and the sensitivity to AsiDNA or olaparib treatment. It is possible that the tumor vasculature would differ between the responding and non-responding tumors, allowing in the first case a better distribution of the drugs. Alternatively, the hypoxic and necrotic content of the tumors could influence their fate during treatment. Further experiments would be required to address this question.

The use of animal models to anticipate adverse effects induced by anticancer treatment has been controversial. In this study, we succeed to recapitulate observations reported in patients following carboplatin treatment. Animals showed neutropenia and myelosuppression typical of platinum toxicity in patients. In a general way, the current study did not reveal any toxicity of the single treatments with olaparib or AsiDNA. The cyclic treatment with olaparib was well-supported by the animals without inducing neutropenia as often observed in patients receiving a daily treatment with olaparib. This tolerance of olaparib could be attributed to the administration protocol that allows a 1-week recovery period without olaparib between weeks of daily treatments. We did not observe a significant increase in bone marrow toxicity when olaparib was combined with chemotherapy. This result is in contradiction with frequent reports of increased bone marrow toxicity during clinical trials where olaparib is associated with carboplatin compared to carboplatin alone (9). Alternatively, all animals receiving olaparib and carboplatin developed acute hepatitis, limiting dose escalation. Though less frequent than neutropenia, liver toxicity was reported in some patients treated with carboplatin (28). In contrast to olaparib, AsiDNA did not increase the toxicity of carboplatin. There was no toxicity in the liver and the neutropenia induced by carboplatin seemed to be moderately reduced with combined treatment. In previous work, we have shown that blood cells (29) as well as normal breast cells (12) are not sensitive to AsiDNA in single treatment or combined with DNA damaging treatments. Clinical trials have confirmed that AsiDNA is not toxic for healthy tissues (11). The lack of toxicity could be related to the unique distribution of AsiDNA that enter tumor cells preferentially through its cholesterol moiety (29) as metabolism of the cholesterol is increased and LDL receptor is overexpressed in most tumors. Olaparib uptake uses the general route of endocytosis and therefore does not appear to show cell specificity. Another possible explanation for the difference in toxicity of the two inhibitors with platinum lies in their different mechanisms of action. In contrast to olaparib (13), AsiDNA does not affect the cell cycle in normal cells and does not increase the number of damage induced. Though the mechanism responsible for the specificity of AsiDNA in tumors is not fully understood, we have observed that its activity correlates with genetic instability, micronuclei formation, and deregulation of cell cycle control often observed in tumor cells. Normal cells could escape to deleterious effect of the inhibition of DNA repair by AsiDNA by blocking division without activating death programs up to disappearance of AsiDNA and restart of DNA repair. The bulky DNA adducts generated by platin are mainly repaired by the NER pathway. There is no clear evidence that olaparib or AsiDNA interferes with NER. However, if NER fails, DNA adducts are converted to DSBs and then can be repaired by the DSBS repair pathways (HR, NHEJ, and MHEJ). Moreover, carboplatin also induces interstrand crosslinks (5% of the total DNA lesions) that are highly cytotoxic and mainly repaired by HR (30). NHEJ seems to play a major role in generating the genomic instability and cytotoxicity in HR-deficient cells treated with PARP inhibitors (31). In HR-proficient cells treated with carboplatin, NHEJ is the main repair pathway involved, and in olaparib-treated healthy tissues, it could induce genomic instability and eventual lethality through its error-prone activity. The fact that AsiDNA has broad inhibitory activity acting on both HR and NHEJ could prevent such deleterious repair to occur in healthy tissues.

This study demonstrates that platinum-resistant tumors derived from the MDA-MB-231 cell line are sensitive to the DNA repair inhibitors, olaparib and AsiDNA. This result suggests that olaparib or AsiDNA treatments could be proposed as monotherapies for patients resistant to carboplatin or who do not tolerate the adverse effects of platinum. Addition of carboplatin to these DNA repair inhibitors has a moderate effect on survival with a better gain for AsiDNA (37%) compared to olaparib (14%). However, resistance to platinum is a process that might appear during treatment and would be difficult to anticipate. Using carboplatin combination with the repair inhibitors before the appearance of resistance could improve efficacy and delay the onset of resistance. Combination of carboplatin with AsiDNA is a promising therapy as it seems to have low toxicity as compared to platinum combination with olaparib. Moreover, it seems to delay the onset of resistance. Actually, whereas olaparib and carboplatin long-term treatments have a high risk of favoring growth of resistant clones in tumor, AsiDNA monotherapy, thanks to its original mechanism of action, seems to be less prone to such events (32). The combination of AsiDNA with carboplatin prevents the emergence of resistant cells in the chemosensitive BC227 cell line. The analysis of carboplatin-resistant clones shows that these cells have a high sensitivity to AsiDNA that could impair a selective growth of resistant clones when both drugs are used in combination.

Whereas, the toxicity of the carboplatin combination with olaparib does not favor such a protocol, the absence of toxicity of AsiDNA (if confirmed in clinic) and its efficacy on platinum-resistant cells would argue in favor of using a combination with carboplatin in a clinical setting.

## Data Availability Statement

The datasets generated for this study are available on request to the corresponding author.

## Ethics Statement

The animal study was reviewed and approved by Comité d'Ethique en Expérimentation Animale de l'Institut Curie (CEEA-IC) (enregistrement #118) in strict accordance with the European Union guidelines for animal care.

## Author Contributions

NH coordinate the animal studies with the help of M-CL. WJ performed the *in vitro* analysis of resistance with the supervision of FB. ST and NB performed blood cell FACS and analysis. J-SS and MD designed the AsiDNA molecules. NH, PN-H, and MD write the manuscript.

### Conflict of Interest

NH, WJ, M-CL, and FB are employees of Onxeo. MD is a consultant for Onxeo. The remaining authors declare that the research was conducted in the absence of any commercial or financial relationships that could be construed as a potential conflict of interest.

## References

[1] van der Noll R Marchetti S Steeghs N Beijnen JH Mergui-Roelvink MW Harms E Long-term safety and anti-tumour activity of olaparib monotherapy after combination with carboplatin and paclitaxel in patients with advanced breast, ovarian or fallopian tube cancer. Br J Cancer. (2015) 113:396–402. 10.1038/bjc.2015.256 26180927 PMC4522644

[2] Wang S Scharadin TM Zimmermann M Malfatti MA Turteltaub KW de Vere White R. Correlation of platinum cytotoxicity to drug-DNA adduct levels in a breast cancer cell line panel. Chem Res Toxicol. (2018) 31:1293–304. 10.1021/acs.chemrestox.8b00170 30381944 PMC6538392

[3] Wernyj RP Morin PJ. Molecular mechanisms of platinum resistance: still searching for the Achilles' heel. Drug Resist Update. (2004) 7:227–32. 10.1016/j.drup.2004.08.002 15533760

[4] Shahzad MM Lopez-Berestein G Sood AK. Novel strategies for reversing platinum resistance. Drug Resist Update. (2009) 12:148–52. 10.1016/j.drup.2009.09.001 19805003 PMC2789192

[5] Zhou JX Feng LJ Zhang X. Risk of severe hematologic toxicities in cancer patients treated with PARP inhibitors: a meta-analysis of randomized controlled trials. Drug Des Devel Ther. (2017) 11:3009–17. 10.2147/DDDT.S147726 29075104 PMC5648323

[6] Ledermann J Harter P Gourley C Friedlander M Vergote I Rustin G. Olaparib maintenance therapy in platinum-sensitive relapsed ovarian cancer. N Engl J Med. (2012) 366:1382–92. 10.1056/NEJMoa1105535 22452356

[7] Swisher EM Lin KK Oza AM Scott CL Giordano H Sun J. Rucaparib in relapsed, platinum-sensitive high-grade ovarian carcinoma (ARIEL2 Part 1): an international, multicentre, open-label, phase 2 trial. Lancet Oncol. (2017) 18:75–87. 10.1016/S1470-2045(16)30559-9 27908594

[8] Mirza MR Monk BJ Herrstedt J Oza AM Mahner S Redondo A. Niraparib maintenance therapy in platinum-sensitive, recurrent ovarian cancer. N Engl J Med. (2016) 375:2154–64. 10.1056/NEJMoa1611310 27717299

[9] Oza AM Cibula D Benzaquen AO Poole C Mathijssen RH Sonke GS. Olaparib combined with chemotherapy for recurrent platinum-sensitive ovarian cancer: a randomised phase 2 trial. Lancet Oncol. (2015) 16:87–97. 10.1016/S1470-2045(14)71135-0 25481791

[10] Dent RA Lindeman GJ Clemons M Wildiers H Chan A McCarthy NJ. Phase I trial of the oral PARP inhibitor olaparib in combination with paclitaxel for first- or second-line treatment of patients with metastatic triple-negative breast cancer. Breast Cancer Res. (2013) 15:R88. 10.1186/bcr3484 24063698 PMC3979135

[11] Le Tourneau C Dreno B Kirova Y Grob JJ Jouary T Dutriaux C. First-in-human phase I study of the DNA-repair inhibitor DT01 in combination with radiotherapy in patients with skin metastases from melanoma. Br J Cancer. (2016) 114:1199–205. 10.1038/bjc.2016.120 27140316 PMC4891504

[12] Jdey W Thierry S Russo C Devun F Al Abo M Noguiez-Hellin P. Drug-driven synthetic lethality: bypassing tumor cell genetics with a combination of AsiDNA and PARP inhibitors. Clin Cancer Res. (2017) 23:1001–11. 10.1158/1078-0432.CCR-16-1193 27559053 PMC5315641

[13] Dale Rein I Solberg Landsverk K Micci F Patzke S Stokke T. Replication-induced DNA damage after PARP inhibition causes G2 delay, and cell line-dependent apoptosis, necrosis and multinucleation. Cell Cycle. (2015) 14:3248–60. 10.1080/15384101.2015.1085137 26312527 PMC4825575

[14] Prasad CB Prasad SB Yadav SS Pandey LK Singh S Pradhan S. Olaparib modulates DNA repair efficiency, sensitizes cervical cancer cells to cisplatin and exhibits anti-metastatic property. Sci Rep. (2017) 7:12876. 10.1038/s41598-017-13232-3 28993682 PMC5634505

[15] Quanz M Chassoux D Berthault N Agrario C Sun JS Dutreix M. Hyperactivation of DNA-PK by double-strand break mimicking molecules disorganizes DNA damage response. PLoS ONE. (2009) 4:e6298. 10.1371/journal.pone.0006298 19621083 PMC2709433

[16] Quanz M Berthault N Roulin C Roy M Herbette A Agrario C. Small-molecule drugs mimicking DNA damage: a new strategy for sensitizing tumors to radiotherapy. Clin Cancer Res. (2009) 15:1308–16. 10.1158/1078-0432.CCR-08-2108 19190126

[17] Croset A Cordelières FP Berthault N Buhler C Sun JS Quanz M. Inhibition of DNA damage repair by artificial activation of PARP with siDNA. Nucleic Acids Res. (2013) 41:7344–55. 10.1093/nar/gkt522 23761435 PMC3753643

[18] Biau J Devun F Jdey W Kotula E Quanz M Chautard E. A preclinical study combining the DNA repair inhibitor Dbait with radiotherapy for the treatment of melanoma. Neoplasia. (2014) 16:835–44. 10.1016/j.neo.2014.08.008 25379020 PMC4212251

[19] Cuenca-López MD Serrano-Heras G Montero JC Corrales-Sánchez V Gomez-Juarez M Gascón-Escribano MJ. Antitumor activity of the novel multi-kinase inhibitor EC-70124 in triple negative breast cancer. Oncotarget. (2015) 6:27923–37. 10.18632/oncotarget.4736 26314846 PMC4695035

[20] Lee JM Hays JL Chiou VL Annunziata CM Swisher EM Harrell MI. Phase I/Ib study of olaparib and carboplatin in women with triple negative breast cancer. Oncotarget. (2017) 8:79175–87. 10.18632/oncotarget.16577 29108297 PMC5668030

[21] Menear KA Adcock C Boulter R Cockcroft XL Copsey L Cranston A. 4-[3-(4-cyclopropanecarbonylpiperazine-1-carbonyl)-4-fluorobenzyl]-2H-phthalazin- 1-one: a novel bioavailable inhibitor of poly(ADP-ribose) polymerase-1. J Med Chem. (2008) 51:6581–91. 10.1021/jm8001263 18800822

[22] Orta ML Höglund A Calderón-Montaño JM Domínguez I Burgos-Morón E Visnes T. The PARP inhibitor Olaparib disrupts base excision repair of 5-aza-2'-deoxycytidine lesions. Nucleic Acids Res. (2014) 42:9108–20. 10.1093/nar/gku638 25074383 PMC4132747

[23] Kötter A Cornils K Borgmann K Dahm-Daphi J Petersen C Dikomey E. Inhibition of PARP1-dependent end-joining contributes to Olaparib-mediated radiosensitization in tumor cells. Mol Oncol. (2014) 8:1616–25. 10.1016/j.molonc.2014.06.008 25028150 PMC5528607

[24] Pernin V Mégnin-Chanet F Pennaneach V Fourquet A Kirova Y Hall J. PARP inhibitors and radiotherapy: rational and prospects for a clinical use. Cancer Radiother. (2014) 18:790–8. 10.1016/j.canrad.2014.09.002 25441760

[25] Jdey W Thierry S Popova T Stern MH Dutreix M. Micronuclei frequency in tumors is a predictive biomarker for genetic instability and sensitivity to the DNA repair inhibitor AsiDNA. Cancer Res. (2017) 77:4207–16. 10.1158/0008-5472.CAN-16-2693 28588010

[26] Mao Z Jiang Y Liu X Seluanov A Gorbunova V DNA repair by homologous recombination, but not by nonhomologous end joining, is elevated in breast cancer cells. Neoplasia. (2009) 11:683–91. 10.1593/neo.09312 19568413 PMC2697354

[27] Helleday T. The underlying mechanism for the PARP and BRCA synthetic lethality: clearing up the misunderstandings. Mol Oncol. (2011) 5:387–93. 10.1016/j.molonc.2011.07.001 21821475 PMC5528309

[28] Sharma A Houshyar R Bhosale P Choi JI Gulati R Lall C. Chemotherapy induced liver abnormalities: an imaging perspective. Clin Mol Hepatol. (2014) 20:317–26. 10.3350/cmh.2014.20.3.317 25320738 PMC4197183

[29] Thierry S Jdey W Alculumbre S Soumelis V Noguiez-Hellin P Dutreix M. The DNA repair inhibitor dbait is specific for malignant hematologic cells in blood. Mol Cancer Ther. (2017) 16:2817–27. 10.1158/1535-7163.MCT-17-0405 28947503

[30] McHugh PJ Spanswick VJ Hartley JA. Repair of DNA interstrand crosslinks: molecular mechanisms and clinical relevance. Lancet Oncol. (2001) 2:483–90. 10.1016/S1470-2045(01)00454-5 11905724

[31] Patel AG Sarkaria JN Kaufmann SH. Nonhomologous end joining drives poly(ADP-ribose) polymerase (PARP) inhibitor lethality in homologous recombination-deficient cells. Proc Natl Acad Sci USA. (2011) 108:3406–11. 10.1073/pnas.1013715108 21300883 PMC3044391

[32] Jdey W Kozlak M Alekseev S Thierry S Lascaux P Girard PM. AsiDNA treatment induces cumulative antitumor efficacy with a low probability of acquired resistance. Neoplasia. (2019) 21:863–71. 10.1016/j.neo.2019.06.006 31362243 PMC6675950

