# Supplementary material for: Preclinical Studies Comparing Efficacy and Toxicity of DNA Repair Inhibitors, Olaparib, and AsiDNA, in the Treatment of Carboplatin-Resistant Tumors
Source: Front Oncol. 2019 Nov 12;9:1097. doi: 10.3389/fonc.2019.01097 (PMC6861330; doi:10.3389/fonc.2019.01097)
Supplement: Supplementary file 1 [file Data_Sheet_1.pdf]

Supplementary Material

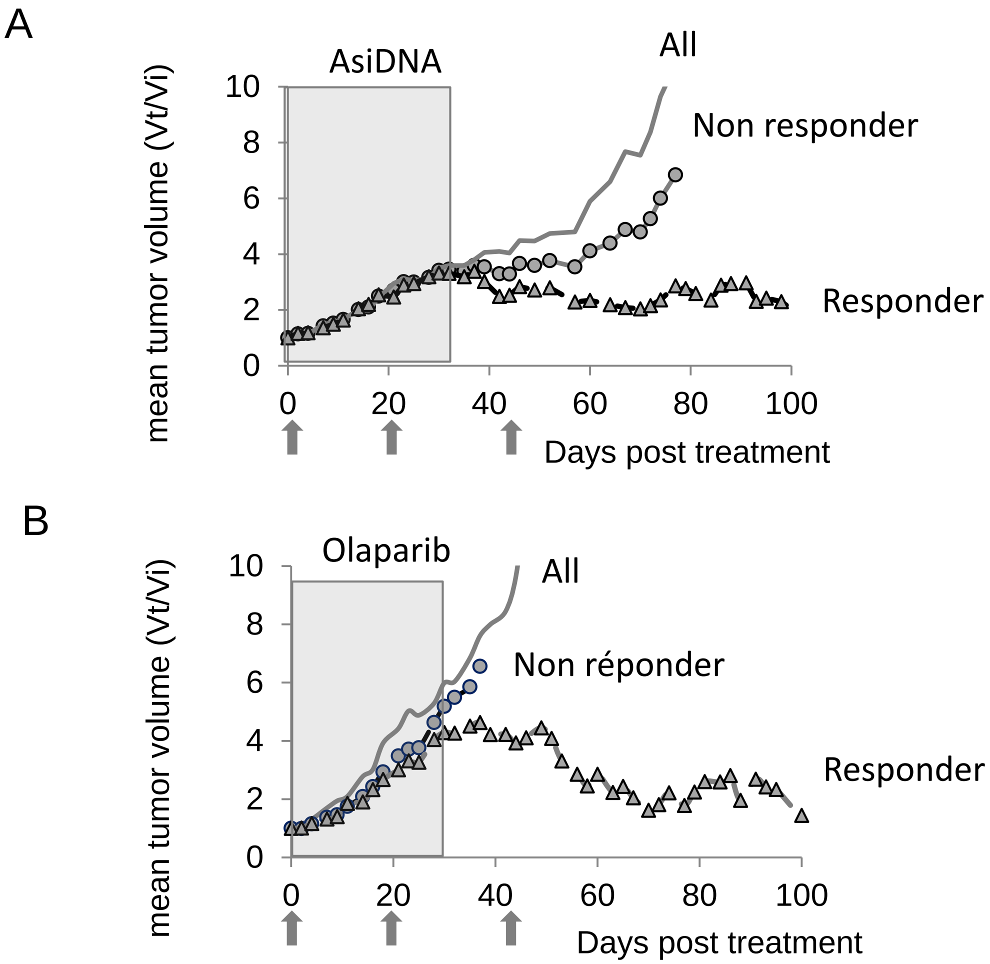

Figure S1: Detailed response to AsiDNA (A) or olaparib (B). Mean tumor growth of all mice (grey line). As response was heterogeneous in both treatments, groups were divided as responder and non responder according to their survival to allow longer growth tumor follow up: responder (A,B, n=4), survival>100 days and non-responder (A,n=4; B, n=5), survival<100). Grey rectangle, progression time before response to treatment; Arrows, beginning of cycles of treatment.

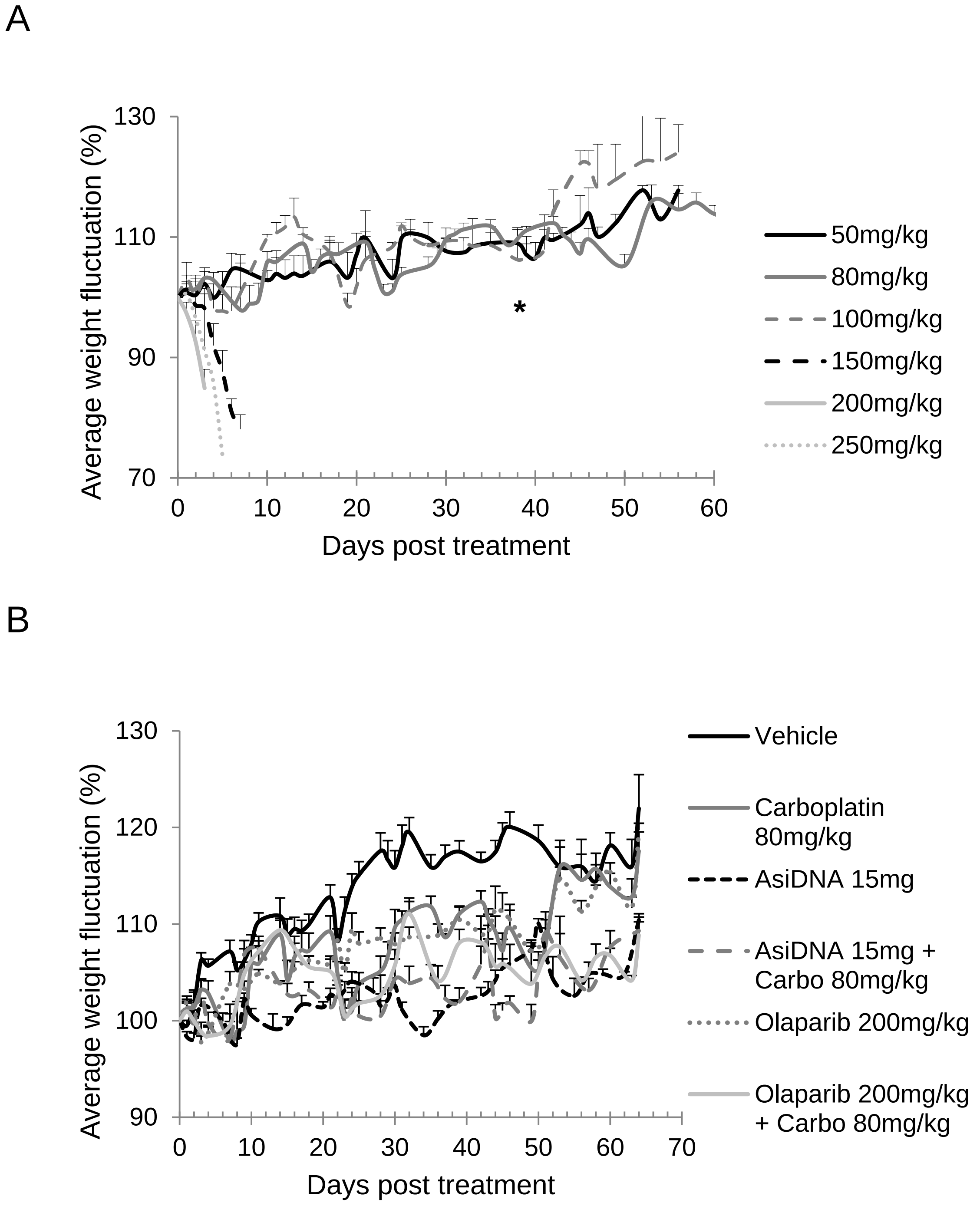

Suplementary figure S2: Average weight fluctuations with various treatments (A) single injection of increasing doses of carboplatin (50, 100, 150, 200, 250mg/kg). *1/3 deaths observed at day 42 following carboplatin treatment at a concentration of 100mg/kg (33% mortality). 100% mortality was observed at carboplatin concentrations of 150, 200 and 250mg/kg. (B) three cycles of combinde treatment with carboplatin (3 x 80mg/kg) with AsiDNA (12 x 15mg) or olaparib (15 x 200mg/kg)

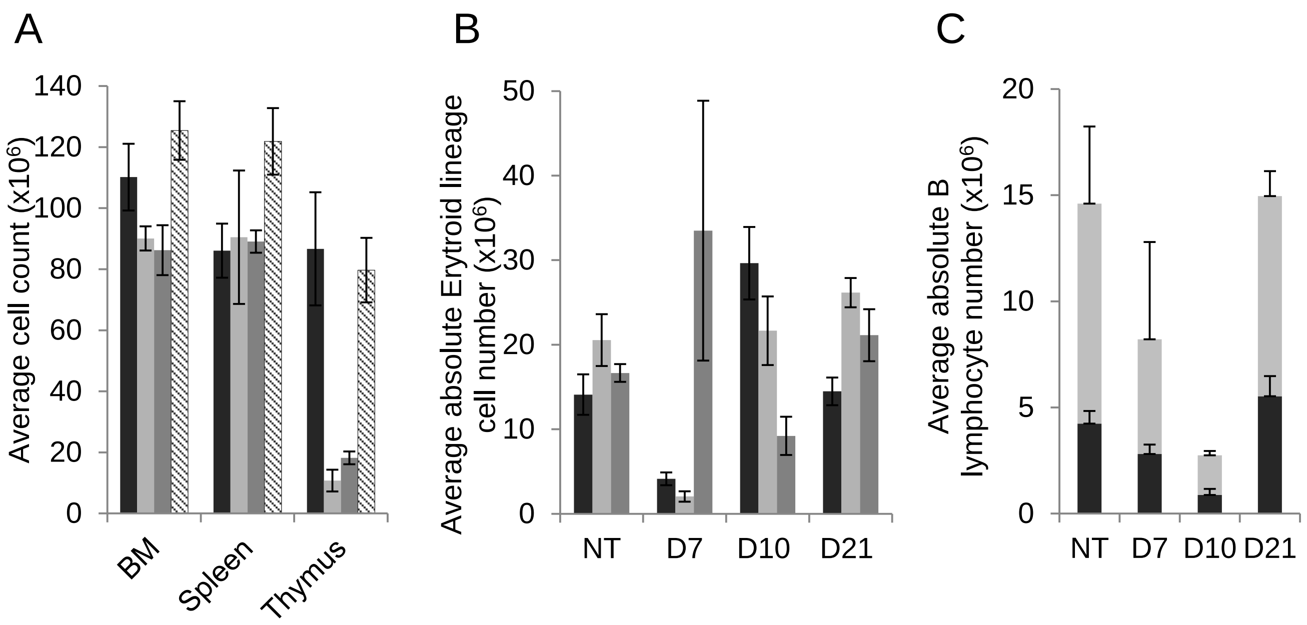

Suplementary Figure S3: Effect of a single administration of carboplatin (80mg/kg). (A) Average total cell counts of bone marrow (BM), spleens and thymus at 0 (black), 7 (light grey), 10 (grey) or 21 (dashed) days post treatment (B) Bone marrow average absolute cell number of the erythroid lineage at 0, 7, 10 or 21 days post treatment : Basophilic erythroblasts (black), Polychromatic erythroblasts (light grey) and Orthochromatic erythroblasts (mid grey) (C) Bone marrow average absolute cell number of Pre/Pro B lymphocytes (grey) and Mature B lymphocytes (black) at 0, 7, 10 or 21 days post treatment.

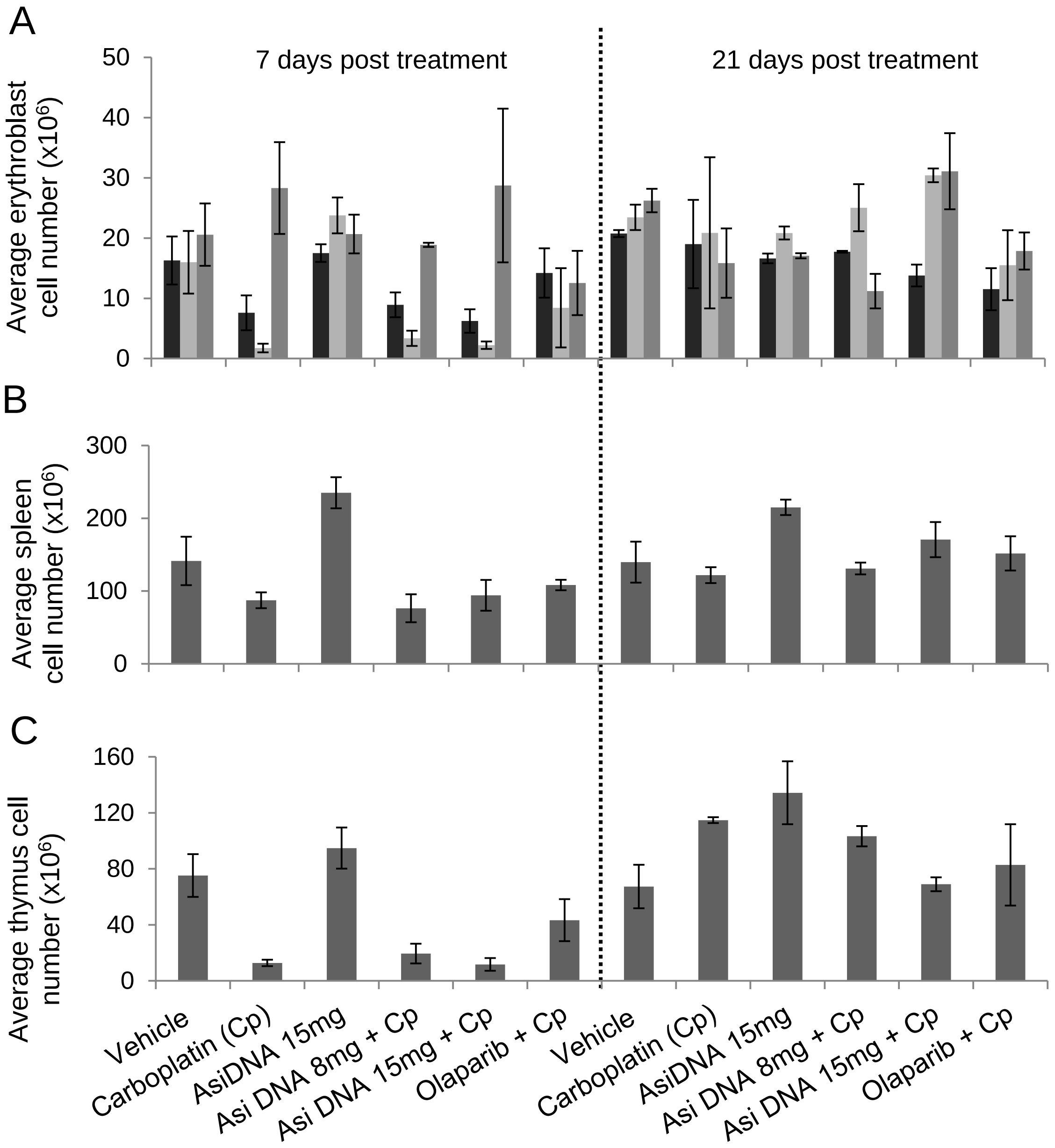

Suplementary Figure S4: Effect of three cycles of different treatments. Different combination of Carboplatin 80mg/kg, AsiDNA 8mg or 15mg and Olaparib 200mg/kg were used to treat mice. Average total cell number 7 and 21 days post treatment of (A) Bone marrow erythroid lineage : Basophilic erythroblasts (black), Polychromatic erythroblasts (light grey) and Orthochromatic erythroblasts (mid grey), (B) spleens and (C) thymus.

Antibody	Manufacturer/catalogue No.	Cell type detected
Rat Anti-mouse CD45R/B220 Hu-PE-clone RA3-6B2	BD Biosciences 553089	Entire B cell lineage (Pre-pro-B cell, pro-B cell, pre-B cell, immature B cell, mature B cell)
Rat anti-mouse CD90.2 (Thy1.2)-FITC-clone 53-2.1	BD Biosciences 553004	T lymphocytes (T cell precursors and mature T cells)
Rat anti-mouse TER-119-PE/erythroid cells	BD Biosciences 553673	Erythroid lineage (erythroid precursors, reticulocytes and erythrocytes)
Rat anti-mouse CD71-FITC	BD Biosciences 553266	Erythroid lineage (erythroid progenitors and precursors)
Rat anti-mouse Ly-6G and Ly-6C (Gr-1) Ms-PE-clone RB6-8C5	BD Biosciences 553128	Granulocytes
Rat anti-mouse CD11b (Mac-1)-FITC-clone M1/70	BD Biosciences 557396	Macrocytes, megakaryocytes, monocytes, activated lymphocytes
PE Rat IgG2b -clone A95	BD Biosciences 553989	Isotype control
FITC IgG2a	BD Biosciences 553456	Isotype control

Table S1: Antibodies used for flow cytometry.

Treatment	Relative risk (p value)	Mice (number)
Vehicle		6
Carboplatin	0.37 (4.3 10-2)	7
AsiDNA	0.25 (4.5 10-3)	7
Carboplatin + AsiDNA	0.14 (3 10-4)	8
Vehicle		6
Carboplatin	0.36 (5 10-2)	6
Olaparib	0.40 (5.2 10-2)	9
Carboplatin + Olaparib	0.20 (3 10-4)	10

Table S2. Efficacy of combination treatments
